# Low body temperature and mortality in older patients with frailty in the emergency department

**DOI:** 10.1007/s40520-022-02098-9

**Published:** 2022-03-01

**Authors:** Janne Alakare, Kirsi Kemp, Timo Strandberg, Maaret Castrén, Jukka Tolonen, Veli-Pekka Harjola

**Affiliations:** 1 Department of Emergency Medicine and Services, Helsinki University Hospital and University of Helsinki, Helsinki, Finland; 2 Department of Geriatric Acute Care, Espoo Hospital, PL 2550, 02070 City of Espoo, Finland; 3 Clinicum, and Helsinki University Hospital, University of Helsinki, Helsinki, Finland; 4 Center for Life Course Health Research, University of Oulu, Oulu, Finland; 5 Department of Internal Medicine, Helsinki University Hospital and University of Helsinki, Helsinki, Finland

**Keywords:** Frailty, Body temperature, Emergency departments, Mortality

## Abstract

**Purpose:**

The aim of this study was to assess the association between low body temperature and mortality in frail older adults in the emergency department (ED).

**Methods:**

Inclusion criteria were: ≥ 75 years of age, Clinical Frailty Scale (CFS) score of 4–8, and temperature documented at ED admission. Patients were allocated to three groups by body temperature: low ≤ 36.0 °C, normal 36.1–38.0 and high ≥ 38.1. Odds ratios (OR) for 30-day and 90-day mortality were analysed.

**Results:**

1577 patients, 61.2% female, were included. Overall mortalities were 85/1577 (5.4%) and 144/1557 (9.2%) in the 30-day and 90-day follow-ups, respectively. The ORs for low body temperature were 3.03 (1.72–5.35; *P* < 0.001) and 2.71 (1.68–4.38; *P* < 0.001) for 30-day and 90-day mortality, respectively. This association remained when adjusted for age, CFS score and gender. Mortality of the high-temperature group did not differ significantly when compared to the normal-temperature group.

**Conclusions:**

Low body temperature in frail older ED patients was associated with significantly higher 30- and 90-day mortality.

## Introduction

Low body temperature is known to be associated with high mortality in acute-care and critical-care patients [1, 2]. In many studies patients with specific acute conditions, such as heart failure or hip fractures, have been found to have a higher risk of death if they are hypothermic at the time of admission [3–5]. In sepsis, low body temperature is associated with higher mortality, but in a recent study, this association did not apply for older patients ≥ 75 years [6].

Low body temperature has been reported to associate with high mortality in acute care, but its significance in the older frail adult group is not yet well described. In this study, we investigated whether low body temperature is associated with mortality in frail older patients in the emergency department (ED).

## Methods

A prospective observational cohort study of frail older adult patients was performed in an academic ED in Finland with some 60,000 adult patient visits per year. Patient inclusion criteria for the study were: ≥ 75 years of age, score of 4–9 on the Clinical Frailty Scale (CFS), and registered resident in the hospital district. Patient visit data were prospectively collected between December 11, 2018 and June 7, 2019. Methods for recruitment and data collection have been described in detail in a previous article [7]. In short, patient data were collected from electronic health records and case report forms.

This secondary analysis included those patients whose body temperature class was documented as part of their initial National Early Warning Score 2 (NEWS2) [8]. According to the ED’s routine protocol tympanic measurement was performed with an automatic digital thermometer. Patients whose CFS score was assessed as nine were excluded, as this group is defined to have a short life expectancy without evident frailty.

The included patients were allocated to three groups based on their NEWS2 temperature class: low-body-temperature group *T* ≤ 36.0 °C (NEWS2 classes ≤ 35.0 °C and 35.1–36.0 °C), normal-body-temperature group *T* 36.1–38.0 °C, and high-body-temperature group *T* ≥ 38.1 °C (NEWS2 classes 38.1–39.0 °C or ≥ 39.1 °C). The outcome measures in this study were 30-day and 90-day mortality.

Age, gender and CFS class were considered as potential confounders and were included in logistic regression analysis.

Background and outcome data were compared between the groups. Statistical significance of the baseline difference between the groups was tested with the chi-square test for binary variables, and with the Kruskal–Wallis *H* test for nonparametric variables. Crude and adjusted odds ratios with 95% confidence intervals (CI) were calculated for outcome data. Binary logistic regression was performed to adjust for potential confounders. A *P* value of < 0.05 was considered statistically significant for all tests. IBM SPSS Statistics version 27 for Windows was used for statistical analyses.

The study was registered at ClinicalTrials.gov on December 20, 2018, identifier NCT03751319.

## Results

Three cases of accidental hypothermia due to environmental exposure and nine cases that had an assessed CFS score of 9 were excluded. A total of 1577 patient visits, 965 (61.2%) female, met the inclusion criteria and were included in the analysis. The median age of the included patients was 85 years with an inter-quartile range (IQR) of 80–89. The median CFS was 6 (IQR 5–7). The measured body temperature was ≤ 35.0 °C for 10 (0.6%), 35.1–36.0 °C for 117 (7.4%), 36.1–38.0 °C for 1339 (84.9%), 38.1–39.0 °C for 89 (5.6%), and ≥ 39.1 °C for 22 (1.4%) of patient visits. Patient characteristics are presented in Table 1.

**Table 1:** Patient characteristics

		*T* ≤ 36.0 °C	*T* 36.1–38.0 °C	*T* ≥ 38.1 °C	*P*
		127	1339	111	
Age	median (IQR)	85 (80–90)	85 (80–89)	85 (79–88)	0.361
Female gender	*n* (%)	69 (54.3)	838 (62.6)	58 (52.3)	0.025
CFS	median (IQR)	6 (4–6)	6 (5–7)	6 (5–7)	0.165
CFS: 4	*n* (%)	34 (26.8)	251 (18.7)	22 (19.8)	
CFS: 5–6		64 (50.4)	691 (51.6)	44 (39.6)	
CFS: 7–8		29 (22.8)	397 (29.7)	45 (40.5)	
*T*: ≤ 35.0 °C	*n* (%)	10 (7.9)			
35.1–36.0 °C		117 (92.1)			
36.1–38.0 °C			1339 (100.0)		
38.1–39.0 °C				89 (80.2)	
≥ 39.1 °C				22 (19.2)	
Death within 30-day	*n* (%)	17 (13.4)	65 (4.9)	3 (2.7)	< 0.001
Death within 90-day	*n* (%)	25 (19.7)	111 (8.3)	8 (7.2)	< 0.001

Significance was tested with the chi-square test for binary variables and the Kruskal–Wallis *H* test for nonparametric data

*CFS* Clinical Frailty Scale, *IQR* inter-quartile range

The outcome data were available for all patient visits. A total of 85 (5.5%) patients died within 30 days and 144 (9.2%) within 90 days of ED admission. During the 30-day follow-up, 17 (13.4%), 65 (4.9%) and 3 (2.7%) patients died in the low-, normal- and high-temperature groups, respectively. During the 90-day follow-up, 25 (19.7%), 111 (8.3%) and 8 (7.2%) patients died in the low-, normal- and high-temperature groups, respectively.

When the low-temperature group was compared to the normal-temperature-group, the crude odds ratio (OR) for 30-day mortality was 3.03 (95% CI 1.72–5.35, *P* < 0.001). When adjusted for age, gender and CFS, the adjusted OR for 30-day mortality was 3.15 (95% CI 1.77–5.61, *P* < 0.001). Crude and adjusted ORs for 90-day mortality were 2.71 (95% CI 1.68–4.38, *P* < 0.001) and 2.84 (95% CI 1.75–4.62, *P* < 0.001), respectively. The high-temperature group had lower mortality compared to the normal temperature group but without statistical significance. Results are shown in Table 2.

**Table 2:** Crude and adjusted odds ratios for 30-day and 90-day outcomes

	OR, crude (95% CI)	*P* for OR, crude	OR, adjusted (95% CI)	*P* for OR, adjusted
Death within 30-day, normal and low body temperature groups				
*T* 36.1–38.0 °C	1		1	
*T* ≤ 36.0 °C	3.03 (1.72–5.35)	< 0.001	3.15 (1.77–5.61)	< 0.001
Age^a^			1.02 (0.99–1.06)	0.235
Female gender			0.71 (0.44–1.12)	0.138
CFS^b^			1.38 (1.13–1.68)	0.001
Death within 30-day, normal and high body temperature groups				
*T* 36.1–38.0 °C	1		1	
*T* ≥ 38.1 °C	0.54 (0.17–1.76)	0.310	0.50 (0.15–1.64)	0.252
Age^a^			1.03 (0.99–1.07)	0.212
Female gender			0.80 (0.49–1.33)	0.399
CFS^b^			1.38 (1.11–1.71)	0.003
Death within 90-day, normal and low body temperature groups				
*T* 36.1–38.0 °C	1		1	
*T* ≤ 36.0 °C	2.71 (1.68–4.38)	< 0.001	2.84 (1.75–4.62)	< 0.001
Age^a^			1.03 (0.99–1.06)	0.113
Female gender			0.87 (0.60–1.26)	0.466
CFS^b^			1.32 (1.13–1.54)	< 0.001
Death within 90-day, normal and high body temperature groups				
*T* 36.1–38.0 °C	1		1	
*T* ≥ 38.1 °C	0.86 (0.41–1.81)	0.690	0.81 (0.38–1.72)	0.581
Age^a^			1.03 (1.00–1.06)	0.075
Female gender			0.82 (0.56–1.22)	0.326
CFS ^b^			1.32 (1.12–1.56)	< 0 0.001

Binary logistic regression model was used for adjusted odds ratios

*OR* odds ratio, *CI* confidence interval, *CFS* Clinical Frailty Scale

^a^One-year increase

^b^One-class elevation

## Discussion

In this study, low body temperature was significantly associated with 30-day and 90-day mortality when compared to normal body temperature in frail older patients in the ED. The association remained after adjusting for age, gender and CFS score. Mortality of patients with fever did not significantly differ from patients with normal body temperature.

This study confirms that the association of low body temperature and mortality applies to frail older adults in the ED. The association may be explained by many different mechanisms: low body temperature may be the result of a more severe condition with multiorgan failure, inflammatory processes, and hormonal factors involving thermoregulation, or low body temperature may directly contribute to unfavourable outcomes [3, 9, 10]. Most older patients visiting EDs, similarly to younger populations, have an acute illness or trauma [11]. Therefore, low body temperature in frail patients within EDs is likely to reflect the same pathophysiological pathways as hypothesised in studies of general acute-care patients with specific illnesses. Besides mechanisms related to the acute condition, low body temperature in ED patients may reflect slowly developed disorders, such as heart failure, in which low body temperature is shown to be associated with rehospitalisation and worse long-term survival [12, 13].

Other longer-term mechanisms may partly explain the findings. Physiological reserves decline in frailty and it is associated with a higher risk of death [14]. In acute care, frailty is associated with higher mortality both in low-acuity and high-acuity groups [15]. Frailty, and related conditions such as malnutrition, sarcopenia and multimorbidity, may lead to slowly diminishing organ functions and low energy metabolism, which can lead to lower body temperature. Therefore, patients with low body temperature may be especially vulnerable subgroup of frail patients with low physiological reserves. We adjusted for the CFS in our analysis, but it is possible that frailty-related low body metabolism is not fully dependent on the observed frailty grade. Low body temperature could help in identifying vulnerable patients who could benefit from geriatric interventions in both acute care and outpatient settings. A longitudinal study would be needed to study this hypothesis.

This study has some limitations. As this was a secondary analysis of a prospective study, the methods were not pre-specified. A larger sample size with more detailed baseline data would have made further adjusting possible; however, for the specific study question, the available data were sufficient. Also, we used the temperature classes of the NEWS2 documentation instead of absolute temperature measures; however, we feel the clinically meaningful cut-off values of the NEWS2 criteria provide adequate categorisation.

## Conclusion

Body temperature is a simple, routine measurement in the ED. Low body temperature in frail older adults in the ED is associated with increased 30-day and 90-day mortality. Further studies are needed to show whether low body temperature is an early independent marker for vulnerability or if it is just secondary to organ dysfunction and temperature dysregulation, and what interventions might improve the outcome in frail patients with low body temperature in an acute-care setting.

## Data Availability

The data are not publicly available or available for sharing due to national juridical restrictions. However, further description or analysis of data are available from the authors upon reasonable request.

## Code availability

Non-applicable.

## References

[1] Ljunggren M, Castrén M, Nordberg M (2016). The association between vital signs and mortality in a retrospective cohort study of an unselected emergency department population. Scand J Trauma Resusc Emerg Med.

[2] Peres Bota D, Lopes Ferreira F, Mélot C (2004). Body temperature alterations in the critically ill. Intensive Care Med.

[3] Payvar S, Spertus JA, Miller AB, Efficacy of Vasopressin Antagonism in Heart Failure Outcome Study with Tolvaptan (EVEREST) Investigators (2013). Association of low body temperature and poor outcomes in patients admitted with worsening heart failure: a substudy of the Efficacy of Vasopressin Antagonism in Heart Failure Outcome Study with Tolvaptan (EVEREST) trial. Eur J Heart Fail.

[4] Uzoigwe CE, Khan A, Smith RP (2014). Hypothermia and low body temperature are common and associated with high mortality in hip fracture patients. Hip Int.

[5] Wiewel MA, Harmon MB, van Vught LA (2016). Risk factors, host response and outcome of hypothermic sepsis. Crit Care.

[6] Shimazui T, Nakada TA, Walley KR (2020). Significance of body temperature in elderly patients with sepsis. Crit Care.

[7] Kemp K, Alakare J, Harjola VP (2020). National Early Warning Score 2 (NEWS2) and 3-level triage scale as risk predictors in frail older adults in the emergency department. BMC Emerg Med.

[8] Pimentel MAF, Redfern OC, Gerry S (2019). A comparison of the ability of the National Early Warning Score and the National Early Warning Score 2 to identify patients at risk of in-hospital mortality: a multi-centre database study. Resuscitation.

[9] Rumbus Z, Matics R, Hegyi P (2017). Fever is associated with reduced, hypothermia with increased mortality in septic patients: a meta-analysis of clinical trials. PLoS ONE.

[10] Singer AJ, Taira BR, Thode HC (2010). The association between hypothermia, prehospital cooling, and mortality in burn victims. Acad Emerg Med.

[11] Fry M, Fitzpatrick L, Considine J (2018). Emergency department utilisation among older people with acute and/or chronic conditions: a multi-centre retrospective study. Int Emerg Nurs.

[12] Nallamothu BK, Payvar S, Wang Y (2006). Admission body temperature and mortality in elderly patients hospitalized for heart failure. J Am Coll Cardiol.

[13] Ahmed A, Aboshady I, Munir SM (2008). Decreasing body temperature predicts early rehospitalization in congestive heart failure. J Card Fail.

[14] Kojima G, Iliffe S, Walters K (2018). Frailty index as a predictor of mortality: a systematic review and meta-analysis. Age Ageing.

[15] Pulok MH, Theou O, van der Valk AM (2020). The role of illness acuity on the association between frailty and mortality in emergency department patients referred to internal medicine. Age Ageing.

